# Supplementary material for: Causal interplay between lactose intolerance and gut microbiota: a combined bidirectional Mendelian randomization and in vivo validation study
Source: Front Nutr. 2026 Jun 1;13:1803337. doi: 10.3389/fnut.2026.1803337 (PMC13265576; doi:10.3389/fnut.2026.1803337)
Supplement: Supplementary file 1 [file Data_Sheet_1.zip › supplementary materials/Forward/leave one out cross validation/ebi-a-GCST90027803.finngen_R12_E4_LACTONAS.pdf]

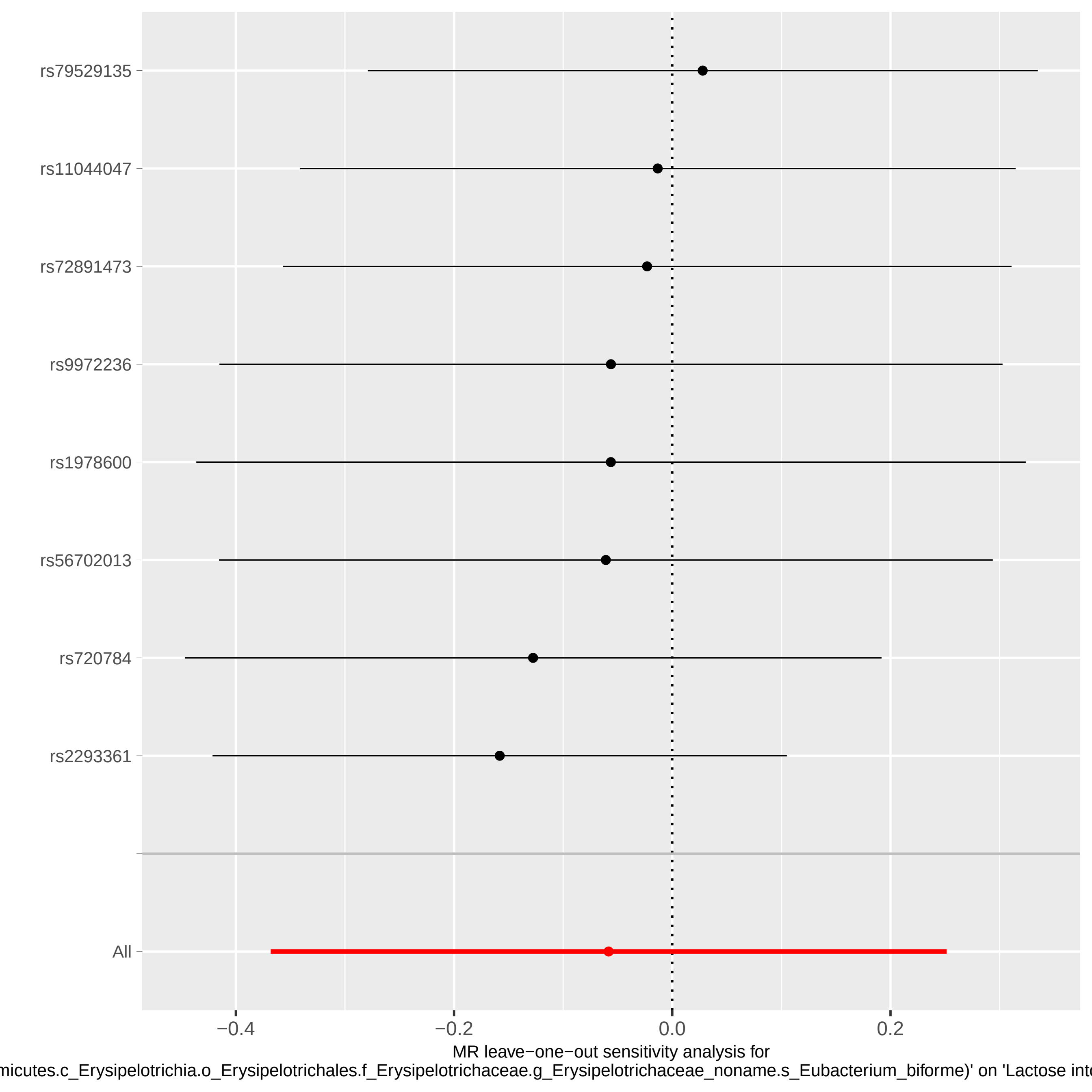
rs79529135
rs11044047
rs72891473
rs9972236
rs1978600
rs56702013
rs720784
rs2293361
All
−0.4
−0.2
0.0
0.2
MR leave−one−out sensitivity analysis for
micutes.c_Erysipelotrichia.o_Erysipelotrichales.f_Erysipelotrichaceae.g_Erysipelotrichaceae_noname.s_Eubacterium_biforme)' on 'Lactose int
